# Advances in Recombinant Adeno-Associated Virus Vectors for Neurodegenerative Diseases

**DOI:** 10.3390/biomedicines11102725

**Published:** 2023-10-08

**Authors:** Leyao Li, Lakshmy Vasan, Bryan Kartono, Kevan Clifford, Ahmadreza Attarpour, Raghav Sharma, Matthew Mandrozos, Ain Kim, Wenda Zhao, Ari Belotserkovsky, Claire Verkuyl, Gerold Schmitt-Ulms

**Affiliations:** 1 Department of Biochemistry, University of Toronto, Medical Sciences Building, 1 King’s College Circle, Toronto, ON M5S 1A8, Canada; 2 Tanz Centre for Research in Neurodegenerative Diseases, University of Toronto, Krembil Discovery Centre, 6th Floor, 60 Leonard Avenue, Toronto, ON M5T 0S8, Canada; 3 Department of Laboratory Medicine and Pathobiology, University of Toronto, Medical Sciences Building, 6th Floor, 1 King’s College Circle, Toronto, ON M5S 1A8, Canada; 4 Institute of Medical Science, University of Toronto, Medical Sciences Building, 1 King’s College Circle, Toronto, ON M5S 1A8, Canada; 5 Centre for Addiction and Mental Health (CAMH), 250 College St., Toronto, ON M5T 1R8, Canada; 6 Department of Medical Biophysics, University of Toronto, 101 College St., Toronto, ON M5G 1L7, Canada

**Keywords:** adeno-associated virus, capsid, gene therapy, gene editing, all-in-one, neurodegenerative diseases

## Abstract

Recombinant adeno-associated virus (rAAV) vectors are gene therapy delivery tools that offer a promising platform for the treatment of neurodegenerative diseases. Keeping up with developments in this fast-moving area of research is a challenge. This review was thus written with the intention to introduce this field of study to those who are new to it and direct others who are struggling to stay abreast of the literature towards notable recent studies. In ten sections, we briefly highlight early milestones within this field and its first clinical success stories. We showcase current clinical trials, which focus on gene replacement, gene augmentation, or gene suppression strategies. Next, we discuss ongoing efforts to improve the tropism of rAAV vectors for brain applications and introduce pre-clinical research directed toward harnessing rAAV vectors for gene editing applications. Subsequently, we present common genetic elements coded by the single-stranded DNA of rAAV vectors, their so-called payloads. Our focus is on recent advances that are bound to increase treatment efficacies. As needed, we included studies outside the neurodegenerative disease field that showcased improved pre-clinical designs of all-in-one rAAV vectors for gene editing applications. Finally, we discuss risks associated with off-target effects and inadvertent immunogenicity that these technologies harbor as well as the mitigation strategies available to date to make their application safer.

## 1. Introduction

Neurodegenerative diseases are a group of disorders that cause progressive damage to the nervous system. As of now, there are no effective cures for these diseases. Adeno-associated viruses (AAVs) have been extensively researched in recent decades as vehicles for the delivery of gene therapies [1].

The goal of most recombinant AAV (rAAV)-based gene therapies that are currently undergoing clinical human trials is to replace or augment the expression of a gene [2,3]. There is also an intense focus on technologies that can reduce the expression of a specific gene or alter the splicing of pre-mRNAs implicated in neurodegenerative diseases [4].

Arguably, the most intriguing but also most challenging disease intervention strategies explore the use of gene editing technology, with clustered regularly interspaced short palindromic repeat (CRISPR)-Cas systems offering exquisite versatility. Examples in this area are recent preclinical in vivo animal studies directed toward correcting progressive vision [5,6] or hearing loss [7]. Despite these advances, so far there are no rAAV-delivered gene editing-based human clinical trials for neurodegenerative disease treatment in the National Institutes of Health (NIH) database. A main reason for this shortcoming is that CRISPR-Cas-based gene editing therapies face additional limitations in efficacy and include risks posed by off-target effects [8] and inadvertent immunogenicity. However, this is a fast-moving field and considerable advances were achieved in the past two years alone.

We have tried to capture these in ten sections in the hope that it will save others the time it takes to keep up with the primary literature. Specifically, Section 2.1 will briefly touch on the history of AAVs. Section 2.2 will introduce current AAVs for the treatment of neurodegenerative diseases undergoing clinical trials. Section 2.3 will explore recent work aimed at improving the tropism of AAVs for brain applications. In addition to describing improved capsids and the methods that were used to identify them, this section will also summarize recent advances in understanding the cellular receptors that contribute to a given tropism. Section 2.4 and Section 2.5 describe the tools and strategies pursued with rAAV vector-based gene therapies. Section 2.6 dissects the payloads of rAAV vectors. Section 2.7 and Section 2.8 introduce ongoing efforts to harness the CRISPR-Cas system for rAAV vector-delivered gene therapies before showcasing recent all-in-one rAAV vectors. Finally, Section 2.9 and Section 2.10 address risks associated with rAAV-based gene therapies before describing ongoing efforts to mitigate these risks. Naturally, because this review covers a broad and highly active research field, it cannot provide the depth that specialized reviews of subtopics can achieve. To address this shortcoming and to keep the bibliography at bay, we have referenced more in-depth reviews that we consider particularly illuminating in the respective sections. We apologize for having had to omit references to many outstanding contributions by our colleagues and for the many instances when we captured the primary works of authors only indirectly by citing review articles.

## 2. Main Section

### 2.1. rAAV Vectors in the Clinic

**Figure 1:**
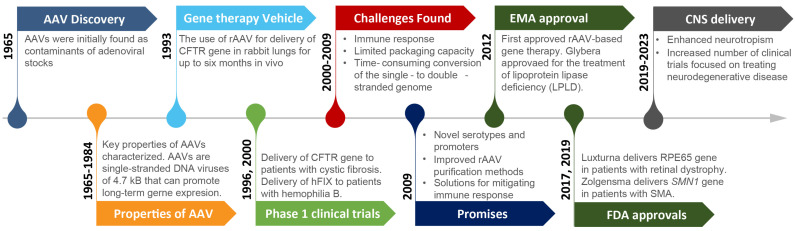
Timeline of discoveries towards deploying AAVs as gene therapy vehicles. The figure focuses on milestone events discussed in Section 2.1 and Section 2.2. Abbreviations: CFTR, cystic fibrosis transmembrane conductance regulator; LPLD, lipoprotein lipase deficiency; RPE65, retinal pigment epithelium-specific protein of 65 kDa; *SMN1*, survival motor neuron 1 gene; SMA, spinal muscular atrophy.

AAVs were initially discovered in 1965 as contaminants of adenoviral stocks (Figure 1) [9]. In the years after, natural AAVs were understood to represent non-enveloped viruses belonging to the genus of *Dependoparvoviruses* within the family of *Parvoviridae*, with a genome size of ~4.7 kilobases (kb). AAVs are surrounded by a 25 nm capsid and need to associate with helper viruses, such as adeno or herpes viruses, to gain access to factors required for their replication. Various characteristics of AAVs, foremost the absence of any disease associated with them and the ability to transduce both quiescent and dividing cells, recommended them as gene transfer vehicles [10,11]. This idea was first put to a test in a 1993 milestone study that used gene-engineered rAAV vectors to deliver a cystic fibrosis transmembrane conductance regulator (CFTR) to the lung epithelium of rabbits [12]. The study validated that rAAV vectors could be used to functionally replace defective genes and that these delivered transgenes can remain active six months post-administration [12,13]. Moreover, the rAAV vector did not alter the growth properties of the host cells and seemed to confer a low risk of insertional mutagenesis [12]. These hopeful conclusions led researchers in the mid-1990s to conduct the first Phase 1 clinical trial based on an intact CFTR gene that was rAAV-delivered to human lungs [13]. This accomplishment was shortly followed by the rAAV vector-based delivery of human factor IX (hFIX) to patients with hemophilia B in the hope of alleviating their bleeding symptoms [14]. Regrettably, these clinical trials had to be abandoned when it became apparent that the rAAV vectors evoked immune responses.

Since then, a lot has been learned about how rAAVs used as gene transfer vehicles in humans can invoke immune responses and encounter neutralizing antibodies [15,16]. The latter occurs due to the widespread human exposure to natural AAV variants that cause approximately 70% of the population to be AAV seropositive.

More than ten years after the first rAAV vector trials in humans, Glybera (alipogene tiparvovec) became the first approved rAAV-based gene therapy (Scott, 2015). Specifically, Glybera was approved by the European Medicines Agency (EMA) in 2012 for clinical use in adult patients afflicted with familial lipoprotein lipase deficiency (LPLD). Glybera’s transgenic expression cassette encodes a human LPL gene variant (LPL^S447X^) that was delivered to patient muscle tissue. One-time administration of Glybera produced sustained LPL^S447X^ expression in muscles for at least seven months. Although this therapy was immunogenic, any impacts on transgene expression remained negligible due to the relative low cytotoxicity of T-cells in muscle tissue [17].

Five years on, the United States Food and Drug Administration (FDA) approved Luxturna (voretigene neparvovec-rzyl) for use in patients with a rare form of inherited retinal dystrophy [18]. Luxturna codes for the retinal pigment epithelium-specific protein of 65 kDa (RPE65) that is mutated in a subset of inherited retinal dystrophy cases [18]. Following administration into the subretinal space of both eyes, clinical follow-up showed that the improvements persisted for more than three years with no serious adverse events or deleterious immune responses [19]. Luxturna’s success can be attributed to several factors, including a perioperative immunomodulatory regimen that involved the steroid prednisone (see also Section 2.10), the removal of empty capsids prior to injection to maximize efficacy and minimize inadvertent side-effects, and the use of a hybrid enhancer/promoter, i.e., cytomegalovirus (CMV) enhancer/chicken β-actin promoter (see also Section 2.6), optimized to strengthen persistent transgene expression [18,19].

### 2.2. rAAV Vectors for Neurodegenerative Diseases in Clinical Trials

In 2019, the FDA approved the rAAV-based gene therapy Zolgensma (AVSX-101, onasemnogene abeparvovec-xioi) for the treatment of young patients afflicted with spinal muscular atrophy (SMA) [20]. Treatment with Zolgensma requires a single intravenous injection of an rAAV vector of serotype 9 (rAAV9) that drives the expression of a functional human survival of motor neuron 1 (*SMN1*) gene [21] from the same hybrid CMV enhancer/chicken-β-actin promoter that was successfully deployed in the Luxturna transfer plasmid. According to the FDA, this is the only approved rAAV-based treatment for a neurodegenerative disease. The rAAV9 capsid was well tolerated, did not evoke a strong immune response, and engaged with several cell-entry receptors [22,23]. Despite these promising initial results, follow-up studies indicated that rAAV9 vector administration may cause elevated levels of detoxifying liver transaminases [24], possibly reflecting compromised liver function. A recent clear-eyed review that took stock of the multi-year efficacy of Luxturna and alternative SMA treatment modalities highlighted complexities, including variations in the treatment responsiveness of patients and inconsistencies in clinical trial infrastructure, leading the authors to conclude that clinical advances to date have extended lives but are far from curative [25].

The preliminary success of Luxturna and Zolgensma trials paved the way for an uptick in rAAV-based clinical trials [26]. They also highlighted a need for mitigating the immune response by considering delivery routes [2,27,28,29,30], optimizing the potency of viral preparations, and augmenting the tropism of rAAV vectors to ensure high specificity for tissues and cells of interest [21,31,32]. Particularly, Zolgensma demonstrated that certain rAAV capsids, including AAV9, can penetrate the blood–brain barrier (BBB) and target the central nervous system (CNS). This precedent fueled a search for further improved serotypes that may be useful in therapies for neurodegenerative disease [28,29,30].

To date, several rAAV vectors designed to target the most prominent neurodegenerative diseases have entered clinical trials. Detailed listings of clinical trials registered in the NIH database (clinicaltrials.gov) were recently published [2,3,33]. A more condensed summary of clinical trial targets and rAAV capsids used for their delivery is included here as an instructive overview of this research landscape (Table 1). In the following paragraphs, we will highlight a subset of these trials in more detail.

The apolipoprotein E isoform (APOE) distribution represents the main risk factor for late-onset sporadic Alzheimer’s disease (AD), with approximately 15% of individuals worldwide who express the polymorphic allele APOE-ɛ4 being at higher risk of disease and those expressing APOE-ɛ2 being somewhat protected [34]. Consistent with this genetic evidence, pre-clinical data in mice established that the rAAV-delivered expression of APOE-ɛ2 can delay plaque formation in mice [35]. The therapeutic potential of a protective APOE allele for delaying AD was corroborated with a case of an individual carrying the presenilin-1 mutation, known to cause early onset AD with high disease penetrance, who was also homozygous for a protective APOE-ɛ3-*Christchurch* R136S mutation [36]. An ongoing LX1001 clinical trial (sponsored by Lexeo Therapeutics) is based on an rAAV serotype originally observed in rhesus monkeys (rAAVrh.10h) [37] which delivers an APOE-ɛ2 transgene following intracisternal (cisterna magna) administration [38].

The loss of dopaminergic neurons in Parkinson’s disease (PD) patients reduces their brain dopamine levels. rAAV2-GDNF is a single-dose AAV2 vector-based treatment developed by Brain Neurotherapy Bio that delivers a functional human glial cell-derived neurotrophic factor (*GDNF*) gene via a bilateral image-guided infusion into the putamen [39]. The expression of exogenous GDNF aims to alleviate PD symptoms by protecting dopamine-producing neurons in the putamen and surrounding brain structures. The treatment (sponsored by Ask Bio and the NINDS) is currently undergoing Phase 1 trials in the US.

AMT130 is a single-dose rAAV5 vector developed by UniQure Biopharma B.V. that is intended to modify the disease course of Huntington’s disease (HD) [40]. The capsid payload for this treatment codes for a microRNA that targets transcripts of the huntingtin (*HTT*) gene for degradation, thereby reducing the expression of the HTT protein. Preclinical investigations established that AMT-130 reduces HTT protein levels and slows the advancement of HD symptoms in animal models. The treatment is currently undergoing Phase 1 and Phase 2 trials in the US and Europe.

The selection of an appropriate delivery route is a critical consideration that determines the efficacy and safety of gene therapies [30,41]. The two main routes for delivering rAAV vectors to brain regions of interest are through surgical intervention or non-invasively through intravenous or intranasal injection [42]. Among the invasive routes, one can distinguish injections directly into the brain parenchyma from injections into the cerebrospinal fluid (CSF), which can be achieved intrathecally, intracisternally, or intracerebroventricularly, with each route being associated with distinct benefits and risks [43,44]. For instance, intraparenchymal injections open the door to delivering rAAV vectors at relatively high titres to a specific region of interest that surrounds the injection site. This can be a benefit if a particular brain structure is to be targeted yet turns into a disadvantage if a broader rAAV vector distribution is desired. Aside from the shared risks associated with surgical intervention in general, including the inadvertent introduction of viruses or bacteria, intraparenchymal injections may pose higher risks of edema or hemorrhages than injections into the CSF. For non-invasive gene therapies to successfully treat neurodegenerative diseases, the rAAV vector must be able to penetrate the blood–brain barrier (BBB). Currently, invasive brain administration routes remain favored in clinical trials (Table 1) because they can deliver relatively high rAAV vector titres directly to brain regions of interest and avoid rAAV vector exposure of other organs, thereby lowering risks associated with peripheral immune responses and inadvertent toxicities [45] (see also Section 2.10). Unsurprisingly, there is an enormous desire for novel rAAV capsids that can safely target the brain through less invasive routes [45,46].

### 2.3. rAAV Capsids with Improved Tropism for Brain Indications

The AAV capsid is comprised of sixty members of three types of protein subunits (VP1, VP2, VP3) encoded by the same cap gene, with alternative splicing and translation from different start codons dictating which subunit is formed [47]. The AAV capsid is composed of the VP1, VP2, and VP3 proteins in approximately a 1:1:10 ratio with high variance.

Natural AAV serotypes are grouped into clades whose members share functional and serological similarities [48,49]. Early attempts to identify AAVs for brain applications were based on the identification of serotypes that naturally target the brain [50,51]. These analyses highlighted AAV2 and AAV9, which are found in Clades B and F, respectively, and were originally isolated from human tissues for their ability to penetrate the BBB and preferentially target CNS neurons [23,48]. More recent results based on radiolabeled AAV9 capsids and positron emission tomography imaging documented that intravenously administered rAAV9 vectors do not efficiently cross into the brains of non-human primates (NHPs), with only approximately 1% of rAAV9 particles observed in the brain after 24 h [52]. Despite these limitations, rAAV9 vectors and rAAV2 vectors continue to dominate ongoing clinical trials for gene therapies targeting the brain, a reality that can be ascribed to their broad tropisms and relatively well understood pharmacology alleviating regulatory hurdles (Table 1).

**Figure 2:**
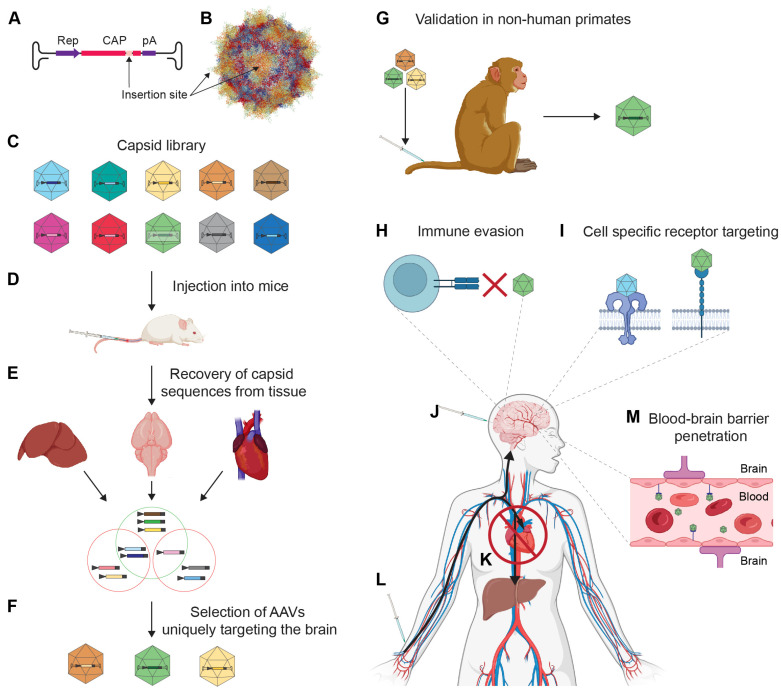
Overview of AAV capsid-directed evolution discovery process for increased brain tropism. (**A**) Randomized insertions are made within the ORF of the cap gene. (**B**) The insertions correspond to positions known to give rise to protrusions within the assembled rAAV capsid; for example, they may target the inner face of 3-fold protrusions shown in yellow. (**C**) The library is assembled in HEK293T cells. (**D**) This library is then intravenously injected into mice. (**E**) Once the virus has been systemically distributed within the body and transduction of cells has occurred, the capsid sequences of rAAV vectors are retrieved from organs of interest and characterized by PCR and next generation sequencing. (**F**) Only sequences that uniquely targeted the brain are selected for downstream use as vehicles for delivering rAAVs to the CNS. (**G**) The selected rAAVs are reassembled, then validated through injection into NHPs. (**H**) In addition to tropism, capsids can be optimized for immune evasion. (**I**) Alternatively, rAAV vectors can be selected for binding to specific receptors. (**J**,**K**) Properties of interest of rAAV vectors are dependent on the downstream application. If the intended application is based on (**J**) intracerebral or (**L**) intravenous injection. For instance, rAAV vectors intended for intravenous therapy are assessed for their ability to de-target other organs, such as the (**K**) liver or heart. (**M**) A major consideration in choosing the delivery route is the ability of a capsid to penetrate the BBB. The figure was assembled with Biorender (https://app.biorender.com/illustrations/6360a504d90edadbfb595fc5, accessed on 1 July 2023).

The main objectives of rAAV capsid engineering are to optimize the tropism of natural AAVs for specific applications and to minimize the natural immune response they invoke (Figure 2) (see also Section 2.10) (see [53] for a recent in-depth review). A common capsid engineering site maps near residue 588 (VP1 sequence) at the inner face of threefold capsid protrusions (Figure 2B). Insertions of amino acids in this region were first used to disrupt the natural heparan sulfate proteoglycan (HPSG) binding site of AAV2 [54]. Subsequent studies demonstrated that insertions at the corresponding site within the cap gene of AAV9 were also able to re-direct the virus despite it lacking a natural HPSG binding site [55], presumably due to the respective sequences forming capsid surface protrusions, not just in AAV2 but also in AAV9. More recently, the rational design of rAAV9 capsids has led to the discovery of AAV.CPP.16 and AAV.CPP.21 (based on single-letter amino acid sequence insertions of TVSALK and TVSALFK, respectively), which exhibit enhanced CNS tropism upon systemic administration in mice and NHPs [56].

Rational rAAV capsid design strategies have, in recent years, largely been replaced by directed evolution techniques, which offer higher throughput [57] and allow many modified capsids to be screened simultaneously [58,59,60,61,62,63,64,65].

An early elegant implementation known as Cre recombinase-based AAV-targeted evolution (CREATE) produced the popular AAV-PHP.B capsid through the randomized insertion of seven amino acids between amino acids 588 and 589 of the AAV9 VP1 gene [58]. Specifically, the randomized transfer plasmids carried a PCR primer recognition sequence flanked by lox sites. Following the injection of the rAAV capsid library into transgenic mice that expressed Cre recombinase in astrocytes, the brain tissue of these mice was harvested. A subsequent PCR analysis led to an enrichment only for the subset of randomized cap sequences that had facilitated the respective rAAVs to enter astrocytes. This is because only in those cells did the Cre-based recombination of lox sites reconstitute the PCR primer orientation required for amplification [66].

The subsequent refinement of the seven amino acid sequence of AAV.PHP.B gave rise to capsids with enhanced specific tropisms, including AAV-PHP.eB and AAV-PHP.S, which target the CNS and peripheral nervous system, respectively [59]. The factors that govern the tropism of AAVs are increasingly understood to be more intricate than originally thought [53]. For instance, a recent study documented that the enhanced brain penetrance of rAAV-PHP.eB, relative to AAV9, depended on the route of administration and strain of mouse tested, with AAV-PHP.eB leading to enhanced transduction of brain cells when it was intravenously injected into C57Bl/6J mice but not B6C3 mice [67]. The differential outcome was subsequently explained by differences in the expression of the Ly6a receptor, which was deemed responsible for the BBB penetrance of the respective AAVs in these mouse lines [68].

A subsequent landmark study built on the AAV-PHP.eB capsid utilized an updated multiplex CREATE (M-CREATE) platform to screen variants in which amino acids 452–458 within VP3 were randomly replaced. This led to the discovery of several AAV capsids with further augmented tropism for brain applications, including AAV.CAP-B10 and AAV.CAP-B22 [65]. Another AAV capsid screen used a design that inverted the main two components of the CREATE platform [62]. Thus, instead of relying on Cre^+^ transgenic mice and lox sites on the transfer plasmid, this system places the gene coding for Cre on the transfer plasmid upstream of a randomized cap sequence library and the lox sites within a transgene cassette that is embedded in the genome of the mice. More specifically, the mice express a loxP-flanked STOP cassette upstream of a fluorescent reporter gene. This ensures that the reporter gene is only expressed in cells that are successfully transduced. Using this model and an iTransduce capsid library of 21 nucleotide insertions at the 588 VP1 insertion site, the discovery of the AAV-F capsid with significantly augmented transduction of astrocytes and neurons was made [62]. A follow-up study demonstrated that AAV-F also robustly transduces the spinal cord [69].

To enable easy identification of members of a library tested, unique nucleotide sequence, barcodes can be added to transfer vectors. An example of this is the barcoded rational AAV vector evolution (BRAVE) approach [61]. Unlike the previously discussed methods, this technique creates a library of rAAVs in which each virus expresses an AAV2-derived cap protein with a 17–22 amino acid peptide insertion after residue N587. The respective peptides were derived from proteins of known function and each transfer vector was barcoded, allowing the parallel identification of all capsid designs that successfully transduced a cell type of interest through next generation sequencing (NGS). Using this library, the authors uncovered that capsids displaying a herpes simplex virus (HSV)-derived peptide (AAV-MNM004) (VMSVLLVDTDATQQ insertion) or a caveolin-2-derived peptide (AAV-MNM008) (SFTSPLHKNENTVS insertion) conveyed dramatically improved retrograde neuronal transport relative to the AAV2 capsid [61]. In a follow-up paper, the same group made use of the AAV-MNM008 capsid to map the neuronal connectivity of transplanted dopaminergic neurons [70].

Machine learning has shown promise in the field of rAAV capsid engineering. Obvious applications are to use this technology to prune libraries of candidate capsids that are unlikely to be viable [71,72,73] or to select for desired traits such as immune evasion [74]. One notable example of this was the discovery of Anc80L65, which in silico modeling had proposed to represent a possible evolutionary ancestor to the current endemic AAVs [75]. In mouse models, Anc80L65 demonstrated greater diffusion and transduction efficacy relative to AAV9 after intravenous injection, though greater transduction was also observed in the liver as well [76]. Recent work involving this vector investigated its use for the treatment of hearing disorders and showed promising results in NHP models [77].

Distinct AAV capsids gain entry into cells and cross the BBB by binding to different receptors and co-receptors on the surface of target cells [78]. This initial contact with the cell surface may involve heparan sulfate proteoglycans and/or N- or O-linked sialic acids as primary cell surface docking sites and various cell surface proteins, including epidermal growth factor receptor, platelet-derived growth factor receptor, fibroblast growth factor receptor 1 (FGFR1), laminin receptor, and alphaV-beta5 integrins as secondary receptors [53,79]. AAV-PHP.B and AAV-PHP.eB capsids have been shown to engage in dynamic interactions with the cell surface glycosylphosphatidylinositol-anchored protein lymphocyte antigen 6 complex locus A (LY6A) [68,80,81]. Once taken up by the host cell, a process that most often appears to rely on endocytosis, a majority of rAAV vectors have been shown to interact with a 150 kDa protein KIAA0319L, now often referred to as the AAV receptor (AAVR) [82]. Atomic resolution cryo-EM data have revealed this interaction to rely on AAVs binding to the second of five polycystic kidney disease repeat domains (PKD2) within AAVR [83,84]. Intriguingly, AAVR is primarily located in the Golgi and so is GPR108, another AAV-interacting protein that appears to be critical for transduction [85], thereby corroborating mounting evidence in support of the conclusion that AAVs transition through this compartment during their journey to the nucleus. The details of how AAVs escape into the cytosol have remained murky, but this step is a prerequisite for their subsequent nuclear translocation through the nuclear pore complex.

Regrettably, it is increasingly apparent that advances in the tropism of AAVs reported in mice translate poorly to NHPs [67,86]. Critical species-specific differences have come to light; for example, the Ly6a gene family, which comprises eight genes in mice and appears to have been critical for the BBB penetrance of AAV capsids selected in this rodent [87], may only consist of one recently discovered distant ortholog (LY6S) in humans [88]. Thus, rAAV capsids selected for their superior BBB penetrance and brain cell-specific tropism in mice may not work in primates [89]. Even when comparing NHPs, there are notable differences. For example, the aforementioned AAV.CAP-B10 and AAV.CAP-B22, which had favorable CNS penetrance in marmosets, did not perform better than AAV9 when injected into macaques [90].

One approach to address this shortcoming has been to employ human neurons as a more pertinent paradigm for the initial discovery of useful capsids. Taking this approach, two novel AAV capsid variants, AAV2-NNPTPSR and AAV9-NVVRSSS, were identified with in vitro differentiated cortical neurons derived from induced pluripotent stem cells (iPSCs) [91]. These capsids exhibited the anticipated improved transduction of human neurons relative to their respective parent AAV2 and AAV9 capsids. Such a cell-based screen may have merit for optimizing the cell-type specific transduction of AAV capsids earmarked for direct intracerebral injections yet may fall short if BBB penetration is required. The latter caveat can be circumvented with an alternative in vitro assay design based on cells forming a BBB-like barrier when grown on transwell plates [92].

Most recently, the trend is to undertake rAAV selection screens directly in NHPs, preferably in Old World primates. This strategy has led to the discovery of AAV.CAP-MAC [93] and a novel family of AAV9 variants with promising NHP brain tropism, termed the proline–arginine loop (PAL) family, generated by insertions of 7-mer sequence motifs bounded by a proline and arginine at amino acid 588 of VP-1 [90].

Advances that can improve access to the human brain will be beneficial at multiple levels. Notably, the selection of effective capsid designs will allow AAV-based gene therapies to be delivered at lower titres, thereby reducing one of their main risks, namely the inadvertent consequences of triggering an immune response (see also Section 2.10).

Once an AAV capsid has been selected for a clinical application (see [94] for an in-depth review on this topic), thought needs to be given to its payload. Gene therapies can crudely be divided into two groups: those that change the expression levels of a gene product without altering the genome itself and those that impose lasting changes on the genome of cells based on gene editing technology. We will follow this distinction when we discuss these two types of gene therapy designs in the next two sections.

### 2.4. Altering the Expression Levels of Gene Products

The subset of gene therapies that alter the expression levels of gene products do so through gene product suppression or augmentation. To date, the majority of AAV-based clinical gene therapy trials for neurodegenerative diseases have been based on strategies that augment the expression of a gene product (see Table 2). Typically, the aim of these therapies is to restore the expression of a protein suspected to contribute to disease by being available in limiting amounts. Because the rAAV-delivered payload in this scenario codes for a protein that is already known to the host, the immune response it invokes is mostly directed to the rAAV capsid and the single-stranded DNA itself. This limited immune response and the absence of off-target effects recommend this approach for its relative safety.

A more versatile but also more immunogenic approach that is currently being explored in pre-clinical development relies on the deployment of CRISPR activation (CRISPRa) technology [95]. To promote transcriptional activation, CRISPRa directs an endonuclease-dead Cas (dCas) that is fused to a transcriptional activator to genes of interest using a single guide RNA (sgRNA). A recent implementation, termed miniCAFE, showed how all components can be made to fit into a single rAAV vector by fusing a small Cas protein, such as *Campylobacter jejuni* Cas9, to small activators, such as VP64-p65-Rta (VPR) [96]. A major strength of CRISPRa would be its potential to deliver AAVs that can activate several genes concomitantly through the expression of more than one sgRNA [97].

Regrettably, when it comes to the most prevalent neurodegenerative diseases, it is less straightforward to think of endogenous proteins whose augmentation would be beneficial than to identify targets central to the aetiologies underlying these diseases whose suppression would be therapeutic. Several of the gene products undergoing the templated structural conversions—widely considered the key mechanism by which neurodegenerative diseases spread within the brain [98,99,100]—do not appear to be essential for survival in human adults, including the amyloid precursor protein, tau, α-synuclein, and the prion protein. Hence, gene product suppression is considered a promising gene therapy approach for several neurodegenerative diseases.

Anyone intent on employing an rAAV vector-based gene silencing strategy faces a wide range of options, including the consideration for whether to interfere with transcription or translation [101,102]. Transcription can be blocked by directing suitable repressor proteins to the gene of interest. The main appeal of this strategy is that it can be highly efficient because only two alleles of the target gene per cell need to be silenced. Compare this to attempts to silence many transcripts of highly expressed genes. Three main approaches have been pursued to achieve this objective with good specificity and efficacy. Zinc finger proteins (ZFPs) [103] offer the advantage of existing naturally within the human genome, thereby keeping immunogenicity at bay. Each zinc finger of 30 amino acids recognizes a 3–4 bp DNA sequence, thereby keeping the coding sequences of arrays of zinc fingers for binding to unique target sites small overall, e.g., <600 bp for a ZFP that can detect a unique DNA binding site of 18 bp [104]. To date, their widespread use has been limited by the need to re-code and adapt ZFPs to genomic DNA target sequences, which can be challenging and time-consuming. Moreover, until recently, ZFPs could only target sites every ~50–200 bp in a random DNA sequence due to limitations in configurational options [105], but this limitation has been mostly overcome with newer ZFP architectures [106]. Taken together, the benefits of using ZFPs may outweigh the challenges associated with them, and recent impressive proof-of-concept data with cell [107] and animal models [108] targeting HTT and tau, respectively, established ZFP-based silencing to be a promising technology.

Transcription activator-like effector nucleases (TALENs), derived from naturally occurring plant pathogenic bacteria, are conceptually like ZFPs. Their main advantage over ZFPs is that their transcription activator-like effector (TALE) tandem arrays can be more easily engineered to target any DNA sequence of interest [109]. Their main downside relative to ZFPs is their increased immunogenicity and approximately threefold larger size, with each repeat of 33–35 amino acids in length conferring binding to just one of the four DNA base pairs [105].

The third main strategy for transcriptional silencing is based on CRISPR interference (CRISPRi) [95]. Recent implementations of CRISPRi use the same functional components as CRISPRa, except that the transcriptional activator is replaced with a transcriptional repressor. CRISPRi can be adapted for rAAV-delivered gene silencing of a gene of interest so long as all of its functional components are selected to fit the size limitations posed by the viral vector choice [110]. The recent use of rAAV-delivered CRISPRi in mouse hippocampal neurons achieved exquisite silencing of hyperpolarization-activated cation channels (HCN) [111]. Nevertheless, the deployment of CRISPRi poses risks associated with off-target effects, immunogenicity of the dCas enzyme, and pleiotropic effects on the transcription of adjacent genes due to the bulkiness of dCas-transcriptional repressor fusion, which can sterically obstruct stretches of 100 bp beyond the target site [112].

Silencing at the level of translation can be achieved with small double-stranded RNAs or single-stranded DNAs. When small RNAs are used, the technology relies on a host-encoded system known as RNA interference (RNAi), which can be triggered by a variety of small double-stranded RNAs known as micro RNAs (miRNAs), short hairpin RNAs, or small interfering RNAs (siRNAs) [113]. Whereas the 21–23 nucleotide siRNAs can be considered the mature product, the longer gene-encoded shRNAs and miRNAs need to first be transcribed from non-protein-coding genes, form short RNA duplexes, and undergo trimming by the ribonuclease dicer before they form siRNA-like double-stranded RNAs. Once in this form, each of these RNAi products gets incorporated into the RNA-induced silencing complex (RISC) that scans mRNAs it encounters for identical or similar sequences. The outcome of this search governs the consequence, with a perfect sequence match causing cleavage of the target mRNA with good specificity and an imperfect sequence match most often leading to a mere repression of target sites [114].

The labile nature of double-stranded RNAs remains a hindrance to the use of siRNAs for the treatment of brain diseases. Although this particular limitation might be overcome with AAV-delivered gene silencing of short-hairpin RNAs (shRNAs) [115], the latter is not trivial either due to the observation that the supraphysiological expression of shRNA silencing precursors can cause toxicity by competing with the processing and function of endogenous miRNAs [116]. This toxicity can be mitigated by driving the expression of shRNAs from cell-type specific Pol II promoters, as opposed to the more broadly expressing Pol III promoters [117,118]. The challenges are in the detail though, as documented by a recent study which established that an AAV-delivered RNAi therapy elicited a toxic neurological response in NHPs due to 3′ inverted terminal repeat (ITR) promoter activity [119]. Therefore, a careful adjustment might be required with this technology to find the right balance between maximizing target engagement yet preventing excessive expression of the small RNA used for silencing. Additionally, the short hairpin DNA structure encoding the shRNA was shown to cause truncated AAV genomes during vector production, which can compromise vector genome homogeneity [120].

This review would be remiss if it failed to mention that, in recent years, suppression at the level of translation for brain applications is most often attempted with single-stranded DNAs, known as antisense oligonucleotides (ASOs), that bind to mRNA to promote cleavage by RNaseH1 [121,122,123]. Single-stranded DNAs are less prone to digestion by endonucleases than RNA. This strength of ASOs can be further augmented through the introduction of modifications that increase their biological inertness [123]. Although ASO-based silencing does not rely on AAV delivery, it is mentioned here because its advantages have made ASOs the dominant technology for gene silencing to date. A challenge associated with ASOs remains their low propensity to cross the BBB, thereby requiring invasive methods of delivery. This in turn limits the safe brain concentrations that can be achieved and generates peak and trough effects in treatment regimes administered months apart [124,125]. The recent discontinuation of four Huntington’s disease ASO clinical trials, which had targeted huntingtin transcripts, as well as mixed results from amyotrophic lateral sclerosis (ALS) trials with chromosome 9 open reading frame 72 (C9orf72) or superoxide dismutase 1 (SOD1) transcripts as the ASO targets put a spotlight on nontrivial complexities in the deployment of this technology [126,127,128]. Although the aforementioned SOD1 trial did not meet clinical endpoints, a somewhat hopeful outcome was the observation that the levels of neurofilament light protein, a surrogate marker of ALS disease severity, were reduced in trial participants who were on the treatment. A more hopeful result from a Phase 1 trial targeting tau expression was published just as we were assembling this manuscript. The treatment reduced total CSF tau concentrations to approximately half the levels seen in untreated cohorts when individuals who received four intrathecal ASO injections over a span of three months were followed for another half year. This result is the most promising to date for an ASO that targets an abundant brain protein. Follow up in Phase 2 and 3 trials will be required before broader conclusions regarding the significance of the tau data can be drawn [129].

### 2.5. Gene Editing Approaches

There is an anticipation that gene editing approaches for neurodegenerative diseases may soon progress towards clinical trials. Early implementations of gene editing technologies were mostly based on zinc finger nucleases (ZFNs) [130] or transcription activator-like effector nucleases (TALENs) [131]. These approaches rely on synthetic tandem arrays of ZFPs or TALE domains (Section 2.4) that are fused to a nuclease, frequently the restriction endonuclease from *Flavobacterium okeanokoites* (FokI). Today, the CRISPR-Cas system has mostly replaced these earlier implementations for the purpose of introducing site-specific cuts or gene edits in pre-clinical evaluations [132].

Regardless of which programmable endonuclease is deployed, gene editing approaches most often begin with the introduction of genomic DNA double-strand breaks (DSBs) to leverage host-encoded gene repair pathways in human cells. The two dominant DNA repair pathways for this purpose are non-homologous end-joining (NHEJ) and homology-directed repair (HDR) [133,134]. NHEJ is repair template-independent and directly joins DNA molecules following the recognition of a DSB by the Ku70-Ku80 heterodimer [135]. Specifically, this heterodimer complex holds the DNA ends in close proximity while recruiting nuclease, polymerase, and ligase components necessary for the resection, insertion, and direct ligation of DSBs [133]. NHEJ is relatively efficient and active throughout all phases of the cell cycle, yet is error-prone, causing it to often introduce small insertions or deletions (indels), a feature exploited for gene editing purposes because it can generate a frame-shift in the coding sequences of target genes, leading to their functional knockout [134].

Unlike NHEJ, HDR requires a homologous repair template. Upon introducing the DSB, 5′ DNA ends tend to be resected by nucleases, creating 3′ overhangs, which can be base-paired with the repair template, causing this method to direct precise and largely error-free gap-repairs. HDR exhibits lower efficiency than NHEJ, a limitation that is further exacerbated by the restricted availability of host factors supporting this repair mode during the S and G2 phases of the cell cycle [136].

NHEJ-mediated in vivo genome editing has achieved 40–50% efficiency in mouse models of AD and HD [137]. Reported efficiencies of HDR-based gene edits include the correction of a mutation within the APP gene in 15% of cells [138] as well as an 8–16% efficiency for the HDR-mediated knock-in of a therapeutic AAV-delivered albumin transgene into the liver of mice [139]. The relatively low HDR efficiency is a major bottleneck for bringing precision gene editing to the clinic. To favor a cell reacting to a DSB with HDR over NHEJ, proteins required for NHEJ can be downregulated, the cell cycle machinery can be influenced, or the local environment can be altered, measures that have been shown to boost the relative efficiency of HDR up to threefold [140]. Microhomology-mediated end joining (MMEJ) is yet another alternative pathway to NHEJ that can be activated if the DSB is produced at a site that is flanked by micro homologous sequences [141]. Although MMEJ has been reported to be 2–3 times slower than NHEJ [141], thereby achieving similar efficiencies as HDR [142], it can be useful in cells in which HDR is not active, including brain cells.

Two relatively novel technologies that warrant mention are base editing (BE) and prime editing (PE). BE directs nucleoside deaminase enzymes to specific genomic sites with the help of catalytically dead dCas-sgRNA complexes. BE can also be used to correct disease-causing single nucleotide mutations at the RNA level [143]. The first proof-of-concept study demonstrating that BE can be packaged within a single AAV vector was recently published [144]. Prime editing (PE) relies on a Cas nickase (nCas) that has one of its two catalytic centres for DNA cleavage inactivated. For PE to work, this nCas needs to be fused to an engineered reverse transcriptase, an arrangement that can facilitate insertions, deletions, and corrections of point mutations, including small indels [143]. PE has shown a low propensity to generate off-target effects and higher efficiency than HDR, as recently demonstrated in mouse models of Duchenne muscular dystrophy and Niemann–Pick disease [143]. To date, the AAV-based delivery of PE is limited by the outsized payloads this system demands, which exceed the recommended size of rAAV transfer plasmids. Following trimming of non-essential elements, the PE technology has recently been packaged in a dual AAV system [145].

### 2.6. Payloads of rAAV Vectors

**Figure 3:**
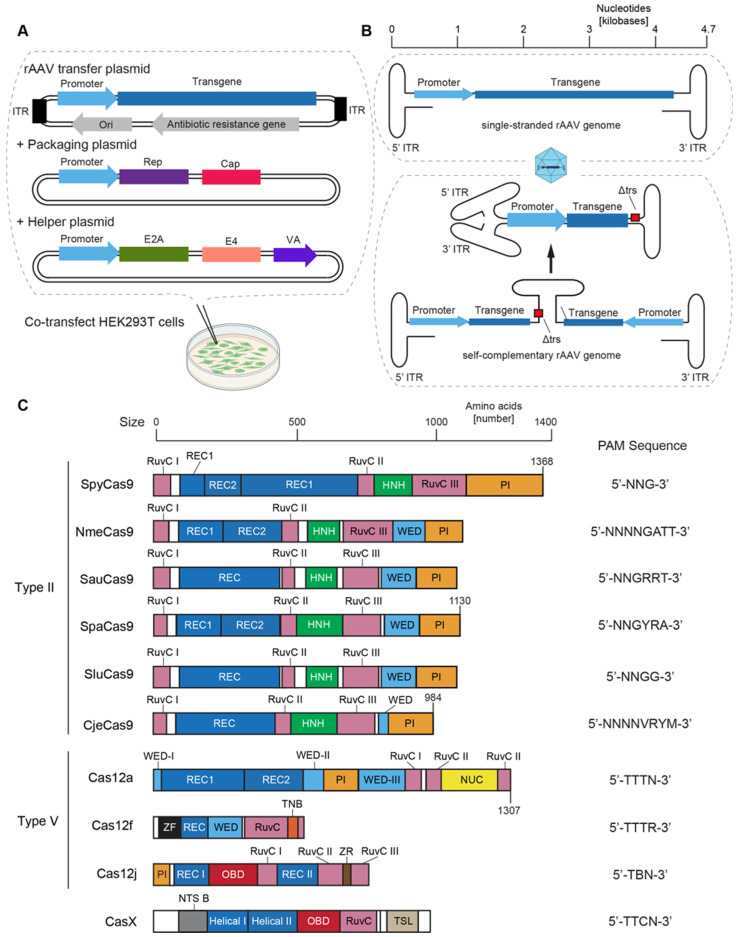
Key elements and domain organization of all-in-one rAAVs. (**A**) Schematic of a generic rAAV transfer plasmid plus the packaging and helper plasmids that need to be co-transfected into HEK293T cells to instruct them how to assemble the rAAV vectors. (**B**) Cartoon depicting the difference between conventional single-strand DNA and self-complementary rAAV genomes. Note that one of the ITRs within the genome of self-complementary rAAVs needs to have its terminal resolution site deleted (Δtrs) to prevent the genome from separating into single-stranded DNA during assembly. (**C**) Domain organization of Cas endonucleases discussed in the text. Cas enzymes and their subdomains are displayed to scale based on their amino acids. Domains that serve the same functions are shown in identical colors. The catalytic domains RuvC and HNH are colored pink and green, respectively. Colors for other domains involved in catalysis are as follows: Nuc, yellow; TNB, brown; TSL, beige; ZF, black; ZR, brown. Nucleic acid-interacting domains are shown in shades of blue (Helical I, Helical II, REC I, REC2, REC III, WED). Remaining domains are as follows: NTSD, grey; OBD, red; and PI, orange. Key to Cas domain acronyms: NTSB, non-target strand binding domain; PI, PAM interacting domain; REC, recognition domain; TNB, target nucleic acid binding domain; TSL, target strand loading domain; WED, wedge domain; ZF, zinc finger domain; ZR, zinc ribbon domain. PAM sequences are shown in single letter nucleotide code. Incompletely specified bases follow the standard nomenclature, i.e., Y, cytosine or thymine; B, cytosine, guanine or thymine; M, adenine or cytosine; R, adenine, or guanine; N, guanine, adenine, thymine or cytosine. (**C**) Domain organization of all-in-one rAAVs discussed in the text that have been employed for in vivo gene editing therapies. The figure was created with Biorender.

The single-stranded DNA genomes of natural AAVs are approximately 4.7 kilobases (kb) in size and are flanked at either end by inverted terminal repeats (ITRs). ITRs are T-shaped hairpin structures formed by the single-stranded DNA that are critical for the expression, replication, and encapsidation of the viral DNA. ITRs also influence the expression of transgenes; for instance, ITRs classified as Class I were reported to generate higher transgene expression than other ITRs [146]. The main genes encoded by natural AAVs are the replication (rep) and capsid (cap) genes (Figure 3A). In rAAVs this rep–cap payload is shifted to a packaging plasmid and the ITR-flanked payloads of transfer plasmids code instead for a promoter, a transgene sequence, and a poly-adenylation signal. The overall size of the recombinant AAV vector genome should be neither too small nor too large, with effective sizes ranging between 4.4 and 4.7 kb. If the payload falls below 2.2 kb, it is advisable to deploy the transgene as a self-complementary DNA [147]. To enhance the efficiency of packaging self-complementary AAV vectors, the so-called terminal resolution site (trs) within one of the two flanking ITRs needs to be deleted (Figure 3B). This prevents the respective ITR from being replicated, thereby blocking the genome from separating into two single-stranded DNA genomes and facilitating the assembly of a double-stranded genome upon transduction [148,149]. Recent studies have shown that the use of self-complementary rAAV vector genomes enables overall improvements in the efficacy that afford dramatic reductions in viral titres [150].

Although rAAV vector genomes that are slightly larger than 4.7 kb can be packaged into rAAV capsids, exceeding the natural size tends to lead to a decrease in transgene expression [151,152,153]. That said, the size tolerances of AAV capsid serotypes can differ considerably, e.g., AAV8 has been shown to handle oversized genomes (as large as 5.73 kb) better than AAV2 [154]. If larger payloads cannot be avoided, dual (or even triple) rAAV vector designs can be considered. Several groups have taken this approach and split the payload into separate sequences that are independently rAAV-delivered and carry overlapping sequences or splice site donor and acceptor sequences for the necessary vector reconstitution in host cells [155,156,157,158]. Despite some proof-of-concept success with the co-transduction of multiple rAAVs, the reality of the asymmetrical transduction of host cells and the differences in their repertoires of factors required for the reconstitution of split transgenes increases variances in transgene expression [159,160]. These shortcomings of co-transduction designs based on multiple rAAV vectors have caused researchers to move towards single-vector all-in-one rAAV vectors [161].

When designing the payload of an rAAV vector, a promoter should be selected that can best ensure transgene expression in the target tissue or cell type of interest. Whereas early studies often made use of the broadly expressing cytomegalovirus (CMV) promoter for efficient transgene expression [162], this promoter is notoriously subject to transcriptional silencing [163]. A popular alternative is the hybrid CMV/chicken beta actin (CBA) promoter, also known by the alternative acronym CAG. This promoter is active in neurons, astrocytes, and oligodendrocytes [164] and has been shown to confer long-term expression, e.g., up to one year of expression in rat brains [165]. A downside of CMV or CAG is that these promoters are also active in peripheral tissues [166]. Consequently, if the goal is to only target a region within the brain or a specific cell type, a promoter exhibiting restricted expression can pre-empt risks associated with off-target effects. Examples include the human synapsin (hSyn), CaMKIIa, and NSE promoters that drive strong neuron-specific expression [166,167], the methyl CpG binding protein 2 (Mecp2) promoter that expresses predominantly in cortical neurons [168], a novel astrocyte-specific GfaABC1D promoter that is considerably smaller than the commonly used GFAP promoter [169], or the murine phosphoglycerate kinase (mPGK) promoter that ensures transgene expression in both cortical neurons and oligodendrocytes [170].

For AAV-delivered gene therapies that rely on the expression of Cas enzymes, additional thought needs to be given to the promoter that controls the expression of single guide RNAs (sgRNA). For this application, undesired off-target effects can be mitigated by working with a broadly expressing Pol III promoter [171]. Human or murine U6 small nuclear RNA promotors [172], the human histone 1 (H1) promotor, or 7SK [150] are commonly used.

Intriguingly, the orientation of a promoter can influence transgene expression [150]. This phenomenon may in some instances reflect the degree of DNA supercoiling: a U6 promoter inserted in the forward direction was observed to give rise to negatively supercoiled DNA upstream of the promoter while a U6 promoter in the reverse direction resulted in positively supercoiled DNA [172]. Ultimately, the selection of the most suitable promoter for a given gene therapy may require at least some iterative evaluations because promoter silencing and crosstalk with other elements of the target cells or rAAV vector may only emerge empirically. For instance, recent studies established unexpected crosstalk between the choice of capsid and the relative promoter activity in various brain cells [173,174].

The expression of any open reading frame (ORF) can be boosted by paying attention to several additional sequence features that impact transcription or translation. Adjustments to the coding sequence that reflect relative abundances of t-RNAs have long been recognized to promote expression. More recently, several studies have put a spotlight on algorithms based on multi-parametric designs (e.g., the GeneArt algorithm) that consider other sequence features to maximize codon and transcript optimization, some of them affording formidable gains in protein expression [175]. One aspect of sequence optimization that is worth paying attention to is the objective to restrict the occurrence of CpG sequences, which are known to trigger the innate immune response (see Section 2.10) [176]. Working with synthetic genes also opens the door to the inclusion of sequence watermarks that can reveal the relative contribution of heterologous versus endogenous genes to total mRNA levels in gene therapy implementations [177]. Whereas the latter application typically leaves little flexibility for altering the protein sequence that is encoded by an open reading frame, there are many opportunities for selecting alternative designs in gene editing applications. Notable in this area are advances in the selection and engineering of Cas proteins that are smaller and optimized for efficiency and on-target specificity (see Section 2.7).

Consideration should also be given to regulatory elements that promote the shuttling of gene products in and out of the nucleus and to poly-A attachment signals. For instance, the inclusion of a woodchuck hepatitis virus posttranscriptional regulatory element (WPRE) increases transgene expression efficiency [178]. Although the precise mechanism through which this benefit manifests is still debated, it might act by promoting transcriptional termination and the subsequent nucleocytoplasmic export of transcripts [179]. Once the protein has been assembled, nuclear localization signals (NLSs), including an NLS derived from the c-Myc protein, can facilitate the nuclear pore complex-mediated import into the nucleus [111]. Often, additional benefit can be attained from the insertion of more than one NLS [180]. Inclusion of a poly-A attachment sequence at the 3′ ends of transgene coding sequences promotes their longevity by reducing their RNAse-mediated degradation [171,180,181].

### 2.7. Optimization of Cas Enzymes for rAAV Gene Editing Vectors

Because the coding sequence of the originally published SpyCas9 is large (~4.1 kb) relative to the AAV carrying capacity, research has shifted towards the discovery of smaller Cas endonucleases (Figure 3C) [182]. Two of the most studied smaller orthologues are the *Staphylococcus aureus* (Sau) and *Neisseria meningitidis* (Nme) Cas9 endonucleases [183,184]. These endonucleases are around 300 amino acids shorter than SpyCas9 yet have comparable editing efficiency [185,186] and relatively low off-target activity [187,188,189]. *Streptococcus pasteurianus* (Spa), *Staphylococcus lugdunensis* (Slu), and *Campylobacter jejuni* (Cje) Cas9 are additional orthologues within this Type II subclass of Cas9 enzymes that exhibit promising editing efficacy [190,191,192]. Each of these shorter Cas9 orthologues have already been deployed for in vivo rodent studies using all-in-one AAV-delivered vectors [31,97,191,192,193,194,195,196,197]. Structural data at atomic resolution [198] have paved a path for ongoing engineering efforts to generate further improved small Cas9-derived nucleases through domain shuffling [199].

Another subclass of Cas endonucleases for rAAV-based gene editing are Type V Cas enzymes that include Cas12a (formerly Cpf1) and are characterized by the presence of a single catalytic domain, RuvC [200]. Although smaller than SpyCas9, Cas12a is composed of 1307 amino acids and is still relatively large [201]. Other Type V enzymes, Cas12f (also known as Cas 14) [202] and Casj, also known as CasΦ [203], were identified from archaea and bacteriophages, respectively, and may be the smallest endonucleases available to date, at almost half the size of SpyCas9. However, unless they are gene-edited, these two orthologues work relatively inefficiently in eukaryotic cells. In addition to their small size, interest in them stems from the observation that they can also work with short PAM sequences, are better than SpyCas9 at preserving genome integrity [204], and have been shown to produce few off-target effects, which may only partially reflect their lower editing efficiencies [205]. Cas12f needs to assemble into an asymmetrical homodimer to generate DSBs, with each protomer fulfilling distinct roles in the recognition and cleavage of the DNA [202]. Convergent evolution has given rise to yet another type of Cas endonuclease, termed CasX, with less than 1000 amino acids that is distinct from Type II and V Cas endonucleases but also exhibits relatively low endonuclease activity [206].

The utility of several of the smallest Cas enzymes is limited by their requirement of extended PAM sequences. To relax PAM recognition limitations, the protein sequences and atomic structures of a subset of Cas endonucleases have been studied for insights that may recommend rational mutagenesis-based alterations of their DNA binding domains [198,207,208]. In addition, the sequences of orthologue Cas endonucleases were screened for naturally occurring variants with altered PAM-interacting domains [194]. Finally, chimeric Cas enzymes, for instance, fusions of SauCas9 and SluCas9, have been created to overcome limitations [209]. Taken together, these efforts have greatly added to the toolbox of Cas endonucleases.

Parallel investigations were aimed at improving the editing efficacy of Type V Cas endonucleases through engineering of variants that enhance binding to mammalian DNA. An example is a report describing the iterative mutagenesis of a natural Cas12f that did not originally function in mammalian cells. The derivative, termed CasMINI, exhibits robust gene editing efficiencies matching those observed with Cas12a [210]. Similar experiments with CasΦ revealed that increases in DNA cutting rates may be accompanied by reductions in target specificity [211]. Structural engineering to enhance the relatively low natural endonuclease activity of CasX or adjust the sequence of the sgRNA has recently led to two derivative genome editors, *Deltaproteobacteria* Cas X (DpbCasX-R3-v2) and *Planctomycetes* CasX (PlmCasX-R1-v2), that exhibited two to three-fold improved catalytic efficiency in human cells [212].

Among the Cas enzymes available to date, SauCas9 has received outsized attention for use in AAV vectors for neurodegenerative disorders. While it is not the smallest endonuclease, SauCas9 is small enough that it can be packaged along with a compatible sgRNA into an all-in-one AAV vector. Moreover, SauCas9 has shown similar editing efficiencies to SpyCas9 [185], more robust editing than several of the smallest Cas enzymes [205], and has been optimized to satisfy a wide target range [207]. Unlike many of the smaller endonucleases, SauCas9 has been validated in several animal models using AAV-mediated delivery, with several studies focusing on gene edits in brain tissue and demonstrating high specificity and efficacy [97,193,195]. For instance, one study used SauCas9 to delete the expression of a gene associated with the onset of Parkinson’s disease-like symptoms in a rat model, documenting the successful mitigation of disease-related deficits [195].

### 2.8. All-in-One rAAV Vector Designs for In Vivo Gene Editing

To date, only a relatively small number of studies have reported pre-clinical in vivo results with all-in-one rAAV gene editing vectors. With the recent discovery of rAAV capsid architectures that exhibit preferential tropism for brain cells (see Section 2.3), the field is poised to apply these leading-edge vehicles for gene delivery to neurodegenerative diseases. In fact, the brain may be well-suited for all-in-one rAAV vector deployment due to its immunologically privileged status caused by a BBB that limits the access of immune cells and other mediators of immune response to the CNS. Moreover, the brain has lower capacity for cellular regeneration than most tissues, which may reduce dilution of episomal rAAV vector genomes, thereby prolonging the efficacy of gene therapies. The following studies were selected to demonstrate the range of tools available today that may see in vivo deployment in gene editing studies of the brain for a wide range of therapeutic applications.

**Figure 4:**
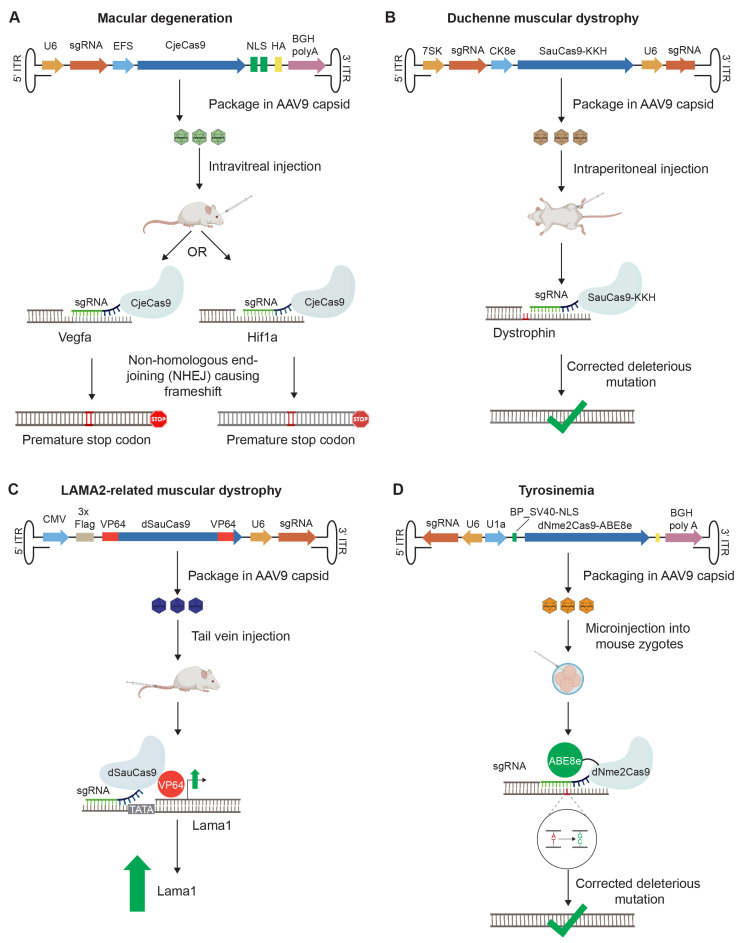
Domain organization of subset of all-in-one rAAV vectors employed for pre-clinical in vivo gene editing therapies. (**A**) Functional knockout of Vegfa or Hif1a genes underlying macular degeneration based on an all-in-one AAV coding for the *Campylobacter jejuni* Cas9. (**B**) Functional knockout of a dysfunctional dystrophin gene causing Duchenne muscular dystrophy with an all-in-one rAAV vector coding for an optimized *Staphylococcus aureus* Cas9. (**C**) CRISPRa-based gene therapy of LAMA2-related muscular dystrophy with an all-in-one AAV coding for a deactivated *Staphylococcus aureus* Cas9 fused to the transcription factor VP64 that promotes upregulation of the *Lama1* gene. (**D**) Correction of tyrosinemia with an all-in-one AAV that codes for a deactivated *Neisseria meningitidis* Cas9 fused to the adenine base editor ABE8e causing it to correct a mutation responsible for this condition. The figure was created with Biorender.

The discovery of Cas9 orthologs of smaller size than SpyCas9 opened the door to all-in-one rAAV-based gene therapy [161]. To our knowledge, the first pre-clinical in vivo study based on this concept transduced an rAAV vector that coded for the small CjeCas9 nuclease of 984 amino acids [191]. The expression was controlled by the elongation factor-1 short (EFS) promoter. The transfer plasmid also comprised a Pol III (U6) promoter-controlled cassette for the expression of an sgRNA that directed the nuclease to cut and generate indels within the Vegfa or Hif1a genes, which are known to contribute to age-related macular degeneration of retinal pigment epithelium cells (Figure 4A).

The formation of indels leads in most instances to frameshifts, which cause the desired ablation of gene expression. The transfer plasmid was packaged into an AAV9 capsid that was administered by intravitreal injection into the eyes of mice. At 42 days post-injection CjeCas9 had generated indels in up to 58% of Hif1a target sites in the retina. Although this advance recommended CjeCas9 for gene editing studies, more recent work has raised concerns that CjeCas9 may be prone to cause cell death by catalyzing non-specific DNA cleavages [213]. Although this non-specific cutting phenomenon can also be observed with SpyCas9 if the nuclease is not saturated with guide RNA, for CjeCas9, guide RNA saturation appears to confer a lesser block to this inadvertent destructive activity.

Several subsequent all-in-one rAAV vector studies have focused on two paradigms with opposite objectives, namely, the correction of disruptions to the ORF of the dystrophin gene, the most common cause of Duchenne muscular dystrophy (DMD) [150,214,215], and the disruption of the proprotein convertase subtilisin/kexin type 9 serine protease (PCSK9) gene that is understood to play a critical role in cholesterol metabolism.

Building on a dual rAAV vector in vivo study, which had set a benchmark for gene therapy of DMD [216,217], two recent all-in-one rAAV designs made use of CjeCas9 or SauCas9-KKH (a derivative with an altered PAM-interacting domain) that were expressed from a synthetic muscle-specific promoter or a creatine kinase 8 (CK8) promoter, thereby restricting the therapy to skeletal muscles and the heart (Figure 4B) [150]. It was previously observed that a single DSB at a specific site can restore dystrophin gene expression with good efficacy. Whereas the first study co-expressed EGFP on the transfer vector, the second implementation aimed to maximize sgRNA levels by concomitantly expressing dystrophin-specific sgRNAs from two distinct Pol III promoters, namely, U6 and 7SK in an all-in-one rAAV. Systemic intraperitoneal delivery of up to 4 × 10^14^ vector genomes/kg into a mouse model of human DMD generated genomic indels in approximately 10% of muscle dystrophin genes, a level that was sufficient to restore 30 to 50% of wild-type expression of the dystrophin gene within one month.

For generating indels in the mouse Pcsk9 gene, one successful in vivo arrangement utilized the U1 promoter to control the expression of NmeCas9, whose nuclear targeting was enhanced by the insertion of nuclear localization sequences [171]. In this implementation, the Pcsk9-gene specific sgRNAs were expressed in reverse orientation from a U6 promoter. The authors documented a >35% in vivo yield of the target gene modification, leading to lower cholesterol levels in mice, and emphasized that the order and orientation of all elements impacted efficacy. In a follow-up manuscript by the same group, three compact orthologs of NmeCas9, termed Nme1–3Cas9, were introduced that utilize other PAM sequences, thereby increasing the possible target density [194]. A follow-up study documented that the compactness of Nme2Cas9 frees sufficient space for the concomitant expression of two sgRNAs or one sgRNA paired with an HDR template to either achieve segmental deletion or precise gene edits, respectively [196]. Finally, separate studies showed that one of two sgRNAs can be used to self-inactivate Cas9, thereby limiting off-target activity or facilitating spatial and temporal control of gene editing activity (see also Section 2.9) [196,218].

Whereas the gene editing designs discussed above relied on Cas nuclease activities, CRISPRa and base editing technology are based on catalytically dead Cas enzymes fused to effector domains. As the field learns to make the best use of the packaging capacity available in all-in-one AAVs, recent reports broke ground showcasing all-in-one rAAV implementations of these space-demanding CRISPR technology derivatives. One report of this kind documented the successful CRISPRa-based upregulation of a disease-modifying gene underlying muscular dystrophy type 1A (MDC1A) [219]. Specifically, the authors rAAV9-delivered an sgRNA targeting the laminin-α1 (Lama1) gene and a dSauCas9 fused to the small VP64 transactivator to promote the transcriptional activation of Lama1, thereby compensating for a disease-causing mutation in the Lama2 paralog and preventing muscle fibrosis and paralysis (Figure 4C) [219]. An advance towards improving the compactness and potency of this technology was the introduction of the MiniCafe transcriptional activator, which is based on the fusion of a truncated VP64-p65-Rta (Vpr) tripartite transactivator to CjeCas9 [96]. Finally, a method termed CRISPReader can free packaging capacity for CRISPRa applications by replacing the promoter with elements that facilitate dSpyCas9-VP64 expression using an ingenious feedback loop. More specifically, the system exploits the inherent basal promoter activity of ITRs to drive the initial expression of a promoterless dSpyCas9-VP64 and an array of sgRNAs embedded within an artificial intron of the dSpyCas9-VP64 gene. Once expressed, the CRISPRa fusion protein is directed to a site adjacent to the TATA box upstream of its own ORF by one of the sgRNAs within the RNA array, thereby boosting its own transcription. Next, another regulatory element, dubbed the RNA activator, which is inserted into the 3′ of the TATA box, recruits factors for the initiation of translation. The system can be incorporated into an all-in-one rAAV vector and additional sgRNAs that target promoters of genes of interest can be included into the RNA array to concomitantly activate the expression of one or more genes of interest [220,221].

The first reports establishing that all-in-one AAVs can be used to deliver size-minimized adenine base editors (ABE) fused to Nme2Cas or orthologs of CjeCas9 designated as Cje2 and Cje3Cas9 were published in 2022 (Figure 4D) [144,180]. Both teams documented the reversion of a disease-causing point mutation in a mouse model of tyrosinemia and studied base-editing of the mouse Pcsk9 gene. In a follow-up report, one of the teams compared the relative efficacy of all-in-one versus dual AAV-based deployment for delivering a size-minimized ABE, showing that the all-in-one solution was 2.5-fold more effective, achieving a 93% knockdown of Pcsk9 in circulation [222]. The authors projected that 82% of all adenines in the human genome should be accessible to base editing if the full spectrum of complementary PAMs of Nme, Sau-KKH, and Nme2 Cas9 variants is exploited.

### 2.9. Off-Target Gene Edits

When undertaking any type of genome edits, it is vital to minimize the risk of off-target effects, which can occur when endonuclease systems are inadvertently directed to genomic sites with similarities to their intended target sites [223]. The consequences of off-target gene edits vary in severity, which spans from harmless mutations of non-coding sequences to severe chromosomal rearrangements that are not only fatal for the respective host cell but can trigger death of the organism if vital functionality is compromised or a cancer is induced. In particular, mutations within highly transcribed chromatin, which often codes for essential genes, may dysregulate cellular homeostasis [223,224]. The use of nickases in conjunction with dual gRNAs could profoundly reduce off-target effects if AAV size limitations and the relatively low efficacy of HDR-based gene editing did not discourage their use [223,225]. To manage this risk and flag the most likely off-target sites, computational methods and in vitro cell-based assays can be employed.

A subset of algorithms for off-target prediction (a comprehensive review can be found in [226]) that can be accessed through web user interfaces are Cas-OFFinder [227], CHOPCHOP [228], COSMID [229], and CRISPR-SE [230]. Recent versions of these in silico tools incorporate machine learning to improve their performance, with the accuracy of their predictions reflecting the volume and quality of empirical data they have been trained on. The functionalities that these algorithms provide are similar but differ in their details. For instance, whereas some algorithms are limited to the prediction of off-target sites based on Cas nucleases, others, including CHOPCHOP, can also flag off-targets of ZFPs and TALENs [228]. DeepCrispr uses a neural network to not only predict off-targets, but also mines epigenetic data to predict whether a given off-target site is accessible [231]. Other algorithms offer complementary functionality with emphasis on optimizing the design of guide RNAs given a specific target. In this category belongs the popular web-based application CRISPOR [232]. In general, it is advisable to apply more than one algorithm and to be alert to the well-validated empirical observation that algorithms have shown limited predictive power for this application. Therefore, it is prudent to complement in silico methods with the validation of off targets using the in vitro methodologies described in the next paragraphs.

Initial in vitro assays for the detection of off-target effects focused on sites that were predicted to have a high propensity to be inadvertently gene edited. Today, unbiased genome-wide assays are available. A conceptually straightforward but inefficient approach could rely on the identification of genome abnormalities by whole genome sequencing. Although feasible, this approach is not recommended due to the high sequence coverage rates required to detect genome alterations that manifest only in a small fraction of cells. Consequently, more efficient methods incorporate enrichment steps for off-target detection, then focus the analysis on the small portion of the genome that is inadvertently modified, thereby achieving deep coverages and detecting gene edits that occur in less than 1:1000 copies of the genome. We will briefly introduce one approach each that is useful for (1) the initial biochemical identification of potential sites, (2) their cell-based refinement, and (3) the in vivo validation of off-target sites. Readers interested in more in depth information are directed to a recent review [233].

One straightforward and popular method for the initial biochemical characterization of gene edits is SITE-seq [234]. Its protocol is initiated by mixing in a test tube a Cas system of interest, an sgRNA, and purified genomic DNA. The in vitro reaction introduces on- and off-target DSBs, whose ends are annealed to biotinylated Illumina-compatible sequence adapters. Next, the whole genomic DNA is fragmented, and a second set of non-biotinylated Illumina sequence adapters are added to the fragments generated by this step. A subsequent biotin-based enrichment retains only fragments that carry Cas-generated ends and, consequently, are biotinylated. These gene edited fragments are amplified by PCR, then subjected to next generation sequencing (NGS) for the identification and subsequent characterization of genome edits.

Several methods have been developed for the in vivo or in cellulo detection of off-targets, with GUIDE-seq representing a useful protocol. To execute a GUIDE-seq analysis, double-strand oligodeoxynucleotides (dsODNs) must be present during Cas cleavage so that this short stretch of sequence gets inserted during the NHEJ-based repair of the DSB. Next, the genomic DNA is mechanically sheared or enzymatically fragmented and Illumina-compatible sequence adapters are added to both ends of each fragment. Using pairs of PCR primers, with one of them targeting the dsODN and the other recognizing the adapter that was added to all genomic fragments, only the Cas gene-edited fragments are amplified and their sequence is determined by NGS.

A method that is useful for validating a suspected Cas genome edit and for characterizing the precise alterations that have been introduced is unidirectional targeted sequencing (UDiTaS) [235]. UDiTaS makes use of tagmentation—a fusion of the terms tagging and fragmentation. More specifically, it exploits the ability of TN5 transposon dimers that are preloaded with barcoded Illumina primers to both fragment the genome and concomitantly transfer the primers to the newly generated genomic ends. Following this step, UDiTaS reveals the precise nature of gene edits within a genomic region of interest by two rounds of PCR amplification and NGS. To this end, a nested PCR design is used, with the first PCR round relying on a target-specific sequence primer with a known overhang. The second round of PCR makes use of this overhang to introduce a second barcoded Illumina sequence primer to amplify and prepare the genomic region that lies between the target-specific sequence primer and the first barcoded primer for NGS. A notable strength of this method is its ability to capture large Cas-induced genomic rearrangements, including deletions, inversions, and translocations that may exceed 100 bps.

Naturally, rather than merely predicting and characterizing off-target edits, a more useful objective can be to preclude their formation. Several strategies have been proposed for this objective, ranging from the use of Cas nickases to the destruction of heterologous Cas proteins once an intended gene edit has been accomplished. One such approach is based on the incorporation of an sgRNA coding sequence and its target site in the transfer vector so that it cleaves the Cas coding sequence, thereby limiting the time available to the nuclease for cutting non-specific target sites. Because nothing prevents the Cas enzyme from becoming activated even during AAV assembly, this strategy is often paired with the expression of anti-CRISPR proteins, whose purpose is to sterically inhibit the respective Cas nuclease from acting during virus production [196,236]. Another method uses rho-dependent transcriptional repression to recruit the Cas nuclease to its own promoter, thereby inhibiting further Cas gene expression [237,238]. Finally, it has been proposed that Cas activity could be turned off through dimerization or allosterical regulation once it is no longer needed [239].

### 2.10. rAAV Vector-Dependent Toxicity and Immune Responses in Humans

Upon rAAV Vector administration for therapeutic purposes, the strength and nature of the immune response will be influenced by characteristics of the host and virus. The main distinguishing feature contributed by the host is if prior exposure to AAVs has occurred, i.e., if the recipient is AAV seropositive, and if the immune system is immature or compromised (through young age, disease, or deliberate manipulation). Consequently, AAV seropositivity is a major exclusion criterion in rAAV vector-based treatment studies to date. The frequency of pre-existing AAV-directed immunity is variable in the global population, depending on one’s race and country of origin, and typically increases with age [240].

Aside from the vector load (10^11^ to 10^14^ vg/kg are common doses) and its method of delivery, critical determinants of the immunogenicity of rAAV vectors are their serotype and payload [241]. The main considerations for the latter are how broadly the transgene will be expressed (a function of the AAV serotype and the choice of the promoter used to drive transgene expression), if the transgene or parts of it are known or foreign to the host, and the integrity of the rAAV vector preparation.

The first line of defense in AAV seronegative individuals is the innate immune response. Even if the capsid does not provoke an immune response, the release of its genomic payload is sure to trigger the innate immune response through the toll-like receptor 9 (TLR9)-based recognition of unmethylated CpG dinucleotides [242,243]. The activated TLR9 receptor then signals through MyD88A, leading to the activation of type I interferons (IFNs). The latter prime CD8+ T cell-dependent adaptive immune responses [242] facilitate the activation of B-cells, thereby leading to neutralizing antibodies and contributing to the activation of cytotoxic T lymphocytes (CTLs) through major histocompatibility complex (MHC) class I-based presentation of capsid- and transgene-derived peptide fragments. The consequence of these steps can be the clearance of transduced cells. Minimally, this would contribute to a loss in transgene expression. In more serious manifestations, the immune response would attack enough healthy tissue to cause death, a fate encountered by a dozen individuals who were enrolled in diverse rAAV vector-based clinical trials during the past ten years (see [244] for details). In AAV seropositive individuals, rAAV vectors would encounter pre-existing capsid-neutralizing antibodies and dormant immune memory from a previous exposure [245].

Although the above only captures the broad strokes of the rAAV vector-induced immune response and the precise molecular underpinnings that govern these events still need to be uncovered, several mitigating steps can be recommended based on these tentative insights. These can be grouped into two complementary approaches that are best pursued in concert, namely, ahead of virus transduction, attempts can be made to outmanoeuvre the immune response, and concomitantly, the immune system can be suppressed.

Among the pre-emptive measures are included the masking of the virus, the engineering of its capsid (see Section 2.3), the selection of an administration route that minimizes immune system exposure [246,247], modifying the T-cell binding domain of Cas enzymes [248], and reducing the presence of unmethylated CpGs.

Shielding of rAAV vectors within exosomes has been reported to increase resistance to capsid-neutralizing antibodies when compared to non-occluded rAAV vector administration [249,250,251], but is not a widely used technique to date. The immune response can also be outmanoeuvered by working with natural AAV serotypes that are not present in humans, such as serpentine AAVs [252] or through engineering that minimizes the antigenicity of the therapeutic rAAV vector. Recent work established that a mere capsid switch may be sufficient to broaden the pool of individuals eligible for enrollment. For instance, by packaging the payload into the rhesus monkey-derived rAAVrh74 serotype, 82% of individuals may be enrolled in a study because they would be seronegative for this capsid [253]. That said, rAAVrh74 is not a serotype that is useful for brain applications. A separate angle for reducing systemic immunogenicity is to facilitate rAAV vector uptake into the brain by promoting capsid interactions with proteins localized at the cell surface of the BBB [254]. To date, approved rAAV vector-based gene therapies in humans are largely based on the monogenic replacement or upregulation of gene products known to the host. Even in this immunologically simpler scenario, rep gene sequence contaminants can find their way into virus preparations and contribute to immunogenicity [255]. For gene editing applications, the expression of Cas enzymes and their derivatives poses additional immunological challenges for human translation.

Arguably, the single feature of rAAV vectors that is most predictive of their capacity for heterologous long-term expression is the presence of hypomethylated CpGs, which allows innate pathogen-associated molecular pattern sensors, such as TLR9, to recognize rAAV-derived DNA as foreign. Two complementary strategies can be pursued to address this challenge: The redundancy of the genetic code can be exploited to eliminate CpGs from all genetic elements within the transfer plasmid that tolerate this step, i.e., mainly the ORFs [256,257]. CpGs that cannot be avoided through synonymous codon replacement can be camouflaged by assembling rAAV vectors in cells that overexpress methyltransferases [258].

Aspects of the immune response not captured by these pre-emptive measures can then be mitigated through immunosuppression. AAV seropositive recipients can be pre-emptively depleted of circulating immunoglobulins using IdeS or Protein M in vivo or plasmapheresis ex vivo. Other immunosuppressive measures typically accompany treatment. For instance, rapamycin is known to target the mammalian target of rapamycin (mTOR) in its regulation of regulatory T-cells (Tregs), thereby maintaining immune homeostasis through the suppression of T-cell activation [259,260,261]. The intraperitoneal co-administration of rapamycin and the steroid prednisolone reduced T- and B-cell activation and released neutralizing serum antibodies against rAAV9 [262]. A similar outcome was obtained when rapamycin administration was combined with the B-cell proliferation inhibitor ibrutinib [263]. A recent study achieved the milestone of rAAV vector re-administration by having generated sufficient immune tolerance through the application of rapamycin-saturated nanoparticles [264].

Despite the many intricacies and challenges associated with rAAV vector-based gene therapies, there is reason to be cautiously optimistic because so far there has been no sign of cancers due to inadvertent genome insertion events in individuals who were treated with rAAV vectors for as long as a decade [265]. Advances in this area are rapid and it is to be anticipated that the use of rAAV vectors for the delivery of effective gene therapies for neurodegenerative illnesses will gain ground. Given the critical role rAAV vectors may play in future personalized medicine, it is critical to tread carefully; so long as a lack of understanding precludes repeat rAAV vector treatments, the focus needs to stay on measures that minimize immune memory. This is to keep the door open for future effective rAAV vector-based treatments in patients who participate in ineffective early rAAV vector therapy trials.

## 3. Conclusions

It is no surprise that the study of rAAV gene therapy vectors represents one of the most innovative and fast-paced fields of research today. If only a fraction of the promises this field offers can be realized, doors would open to the treatment of some of the most intractable diseases, including dementias. For this to happen, formidable hurdles still need to be overcome. Front and center among them are the need to boost the delivery and on-target efficacy of gene therapies, while concomitantly mitigating unintended off-target effects, including toxicity and immunogenicity of the gene therapy vehicles. In contrast, methods for AAV vector production that can provide high purity preparations with excellent yields are no longer a bottleneck in this field; this is why we did not discuss this subtopic, despite considerable innovations continuing to emerge in this domain as well. The past three years have been marked by both remarkable advances and setbacks in AAV capsid designs that favor the targeting of brain cells in NHPs following systemic administration. We have learned that the molecular underpinnings that govern capsid tropism are highly complex and are not only changing over time but can differ within members of the same species. Critically, the most promising capsids with NHP brain tropisms to date have not yet made it into the clinic, and we anticipate that they will have a major impact on bench-to-bedside translation. We are also looking forward to seeing the first therapeutic AAV gene editing vectors for neurodegenerative disease being tested in clinical trials in the next few years. Finally, we are excited about rapid advances in immune-suppressive treatment regimes that may herald a future in which repeat AAV vector administrations can be managed. If this goal can be achieved, it would greatly increase our chances to boost the efficacy of AAV vector-based treatments targeting key neurodegenerative disease proteins that are often widely expressed throughout the brain. The field epitomizes the best in science, where incremental contributions by many have led to astounding advances in the past few years alone. With this review, we hope to have made our own small contribution toward this shared goal and look forward to staying tuned.

## Figures and Tables

**Table 1:** Clinical trials of rAAV-based gene therapies for neurodegenerative diseases.

Therapeutic				Specificity			Trial Benchmarks			
Disease ^1^	Gene (Name)	Protein Function and Therapeutic Rationale	Mechanism/Approach	Delivery	Serotype	Promoter ^2^	Cumulative Duration	Most Advanced Phase	Sponsor	Trial ID ^3^
AD	APOE2 (LX1001)	Apolipoprotein E ε2 is an isoform of APOE that is reduced in neurons in AD and confers protection.	EGE/AE	Cisterna magna	AAVrh.10h	CAG	2019–2023	I	Lexeo Therapeutics	NCT05400330
	hTERT (AAV-hTERT)	Human telomerase reverse transcriptase elongates chromosomal ends in dividing cells. hTERT activity is reduced in neurons in AD.	EGE/AE	Intravenous and intrathecal	unknown	unknown	2019–2021	unknown	Libella Gene Therapeutics	NCT04133649
	NGF (CERE-110)	Nerve growth factor promotes the growth and development of sympathetic and parasympathetic neurons. NGF activity is reduced in neurons in AD.	EGE/AE	Nucleus basalis of Meynert	AAV2	CAG	2004–2015	II	Sangamo	NCT00876863
	BDNF (AAV2-BDNF)	Brain-derived neurotrophic factor promotes the development and maintenance of neuronal populations. BDNF activity is reduced in AD neurons.	EGE/AE	Intraparenchymal	AAV2	unknown	2021–2025	Recruiting	Mark Tuszynski	NCT05040217
PD	AADC (AAV- hAADC-2)	Aromatic L-amino acid decarboxylase contributes to the biosynthesis of dopamine. There is a progressive loss of dopaminergic neurons expressing AADC in PD.	EGE/AE	Putamen	AAV2	CMV	2013–2022	II	Voyager Ther, Neurocr. Biosci	NCT00229736
	GAD (AAV-GAD)	Glutamic acid decarboxylase plays a role in the biosynthesis of GABA. Progressive loss of GABAergic neurons expressing GAD reduces motor inhibition in PD.	EGE/AE	Subthalamic nucleus	AAV2	CBA	2003–2005	I	Neurologix	NCT05603312
	GDNF (AAV2-GDNF)	Glial cell-derived neurotrophic factor promotes the survival of neurons. There is a progressive loss of nigrostriatal dopaminergic neurons expressing GDNF in PD.	EGE/AE	Putamen	AAV2	unknown	2013–2022	I	Ask Bio, NINDS	NCT04167540
	NRTN (CERE-120)	Neurturin is a neurotrophic factor that promotes the survival of neuronal populations. There is a progressive loss of dopaminergic neurons expressing NRTN in PD.	EGE/AE	Putamen	AAV2	CAG	2005–2017	II	Sangamo	NCT00252850
	GBA1 (LY3884961)	Glucocerebrosidase plays a role in the biosynthesis of glucose and ceramide. Aberrant mutations in GBA1 in dopaminergic neurons occur in PD.	EGE/AE	Cisterna magna	AAV9	CBA	2020–2027	I/II	Prevail Therapeutics	NCT04127578
FTD	GRN (PBFT02)	Granulin promotes the growth, survival, and maintenance of neuronal and microglial populations. GRN activity is reduced in neurons in FTD.	EGE/AE	Cisterna magna	AAV1, AAV9	unknown	2020–2027	I/II	Passage Bio, Prevail Therapeutics	NCT04747431
HD	HTT (rAAV5-miHTT)	Huntington is a cytosolic protein that regulates neuronal and glial function. There are aberrant ≥36 CAG repeats in the HTT gene in HD.	EGR using miRNA/AE	Intrastriatal	AAV5	unknown	2019–2026	I/II	UniQure Biopharma	NCT04120493
SMA	SMN (AVXS-101)	Survival motor neuron is a neurotrophic factor that promotes the maintenance of motor neurons. SMN activity is reduced in motor neurons in SMA.	EGE/AE	Intravenous and intrathecal	AAV9	CBA	2014–2035	IV	Novartis Gene Therapy	NCT02122952

^1^ FTD, frontotemporal dementia; HD, Huntington’s Disease; SMA, spinal muscular atrophy; EGE, exogenous gene expression; EGR = exogenous gene repression; AE, augmented expression; SE, supressed expression. ^2^ Note that CBA and CAG are alternative acronyms for the same promoter. In assembling this table, we presented the information on promoters as they were provided by the respective study authors. ^3^ For additional details regarding these trials, see clinicaltrials.gov. In instances when multiple clinical trials have been associated with a gene therapy, only the Trial ID for the most recent trial is shown.

**Table 2:** Characteristics of rAAV-based gene therapy approaches pursued in pre-clinical in vivo studies.

	Objective	Method ^1^	Payload	Target	Strengths	Weaknesses
Altering gene expression	Augmentation	Transgene expression	Functional gene controlled by the Pol II promoter	Heterologous gene	Low immunogenicity	Expression based on non-natural promoter
		CRISPRa	dCas fused to a transcriptional activator (e.g., VP64), with the Pol III promoter driving sgRNA expression	DNA sequence defined by protospacer (~17–24 bases) and PAM (~2–6 bases)	Adaptable and efficient	Large size, off-target sites, immunogenicity
	Suppression	ZFP	Array of zinc finger DNA binding domains	DNA promoter sequence defined by base triplets (typically 18–33 bases)	Small size, low immunogenicity	Complex selection and optimization
		TALE	Array of TALE DNA binding domains	DNA promoter sequence defined by base singlets	Easily sequence adaptable	Larger size and heightened immunogenicity relative to ZFPs
		CRISPRi	dCas fused to transcriptional repressors (e.g., ZIM3) and sgRNA	DNA promoter sequence defined by protospacer and PAM	Adaptable, efficient	Larger size and heightened immunogenicity relative to ZFPs
		RNAi	shRNA expression from the Pol II or III promoter	mRNA transcript defined by shRNA sequence	Adaptable, few off-target sites	Can compete with natural miRNA processing
Gene editing	Knockout	ZFN	ZFPs fused to an endonuclease (e.g., Fok1)	DNA gene sequence defined by base triplets	Low immunogenicity	Low efficiency, non-uniform, knockout
		TALEN	TALE fused to an endonuclease (e.g., Fok1)	DNA gene sequence defined by base singlets	Easily sequence adaptable	Larger size and heightened immunogenicity relative to ZFPs
		CRISPR-Cas via NHEJ/MMEJ	Cas nuclease or nCas nickase and sgRNA(s)	DNA gene sequence defined by protospacer and PAM	Adaptable and efficient	Large size, off-target sites, immunogenicity
	Directed mutagenesis	CRISPR-Cas via HDR	Cas nickase (nCas), plus sgRNA(s) and repair template	DNA gene sequence defined by protospacer and PAM	Accurate large insertions	Large size, off-target sites, non-uniform insertion
		BE	Inactive Cas (dCas) fused to base editor and sgRNA	DNA gene base defined by protospacer and PAM	Few off-target sites, generates precise edits	Large size, low efficiency, only single base edits
		PE	Cas nickases (nCas) fused to reverse transcriptase (e.g., M-MLV) and pegRNA and sgRNA	DNA gene sequence defined by protospacer and PAM	Few off-target sites, generates precise edits	Large size, low efficiency, only for small edits

^1^ Abbreviations: BE, base editor; CRISPR, clustered regularly interspaced palindromic repeats; CRISPRa, CRISPR activation; CRISPRi, CRISPR inhibition; HDR, homology-directed repair; MMEJ, microhomology-mediated end joining; NHEJ, non-homologous end joining; PE, prime editor; pegRNA, prime editing guide RNA; RNAi, RNA interference; sgRNA, single guide RNA; shRNA, short hairpin RNA; TALE, transcription activator-like effector; ZFP, zinc finger protein.

## Data Availability

Not applicable.
